# An exclusionary screening method based on 3D morphometric features to sort commingled atlases and axes

**DOI:** 10.1038/s41598-024-63029-4

**Published:** 2024-06-07

**Authors:** Annalisa Cappella, Andrea Palamenghi, Riccardo Solazzo, Debora Mazzarelli, Daniele Gibelli, Chiarella Sforza, Cristina Cattaneo

**Affiliations:** 1 Dipartimento di Scienze Biomediche per la Salute, Università degli Studi di Milano, Via Luigi Mangiagalli 31, 20133 Milan, Italy; 2 U.O. Laboratorio di Morfologia Umana Applicata, IRCCS Policlinico San Donato, 20097 San Donato Milanese, Italy; 3 LABANOF, Laboratorio di Antropologia e Odontologia Forense, Dipartimento di Scienze Biomediche per la Salute, Università degli Studi di Milano, Via Luigi Mangiagalli 37, 20133 Milan, Italy; 4 LAFAS, Laboratorio di Anatomia Funzionale dell’Apparato Stomatognatico, Dipartimento di Scienze Biomediche per la Salute, Università degli Studi di Milano, Via Luigi Mangiagalli 31, 20133 Milan, Italy

**Keywords:** Comingled remains, Re-association, 3D–3D superimposition, Mesh-to-mesh value, Distance analysis, Virtual anthropology, Virtual anatomy, Anatomy, Anthropology

## Abstract

In forensic commingled contexts, when the disarticulation occurs uniquely at the atlantoaxial joint, the correct match of atlas and axis may lead to the desirable assembly of the entire body. Notwithstanding the importance of this joint in such scenarios, no study has so far explored three-dimensional (3D) methodologies to match these two adjoining bones. In the present study, we investigated the potential of re-associating atlas and axis through 3D–3D superimposition by testing their articular surfaces congruency in terms of point-to-point distance (Root Mean Square, RMS). We analysed vertebrae either from the same individual (match) and from different individuals (mismatch). The RMS distance values were assessed for both groups (matches and mismatches) and a threshold value was determined to discriminate matches with a sensitivity of 100%. The atlas and the corresponding axis from 41 documented skeletons (18 males and 23 females), in addition to unpaired elements (the atlas or the axis) from 5 individuals, were superimposed, resulting in 41 matches and 1851 mismatches (joining and non-joining elements). No sex-related significant differences were found in matches and mismatches (p = 0.270 and p = 0.210, respectively), allowing to pool together the two sexes in each group. RMS values ranged between 0.41 to 0.77 mm for matches and between 0.37 and 2.18 mm for mismatches. Significant differences were found comparing the two groups (p < 0.001) and the highest RMS of matches (0.77 mm) was used as the discriminative value that provided a sensitivity of 100% and a specificity of 41%. In conclusion, the 3D–3D superimposition of the atlanto-axial articular facets cannot be considered as a re-association method per se, but rather as a screening one. However, further research on the validation of the 3D approach and on its application to other joints might provide clues to the complex topic of the reassociation of crucial adjoining bones.

## Introduction

In the resolution of commingled bone assemblages, the assessment of congruence of adjoining bones has been described as one of the most convincing lines of evidence to re-associate portions of skeletons^1–6^. Traditionally, the re-association of articulating elements is performed manually, assessing the degree of congruence of the two mating surfaces. This is therefore a subjective method, whose reliability decreases as the number of subjects involved increases^2^, and the outcome of the association strongly relies on the experience of the observer^7^. Also, as a standalone technique, ‘manual articulation’ may not yield proficient results when it is applied to skeletal portions that do not show a significant area of contact between the articular surfaces^6^.

Researchers have tried to answer the lack of objectivity with osteometric and statistical methods to re-associate articulating bones. Early attempts at developing sound statistical models included the re-association of the hip joint^8,9^. This approach was originally explored by Buikstra and Gordon^10^ that suggested a quantification of the degree of congruence between cervical vertebrae, in the resolution of a forensic case. Over time, osteometric models have been proposed also to re-associate humeri with scapulae and ulnae^11^, hip, knee, and ankle^12,13^, the subtalar joint^14^, and the craniovertebral junction^15^. Despite the great potential, such as cost efficiency and small error rates^16^, osteometric techniques still present limitations that could affect the efficacy. These include the size of the commingled assemblage and the disparity of size among the individuals in the sample^17^. Furthermore, as they are statistical methods, they are closely linked to the reference skeletal population, which makes them hardly applicable to unknown samples^18^. A recent study applied linear measurements to CT-scans images to evaluate the metric relationship between mandibles and skulls, suggesting its exclusionary potential in sorting commingled remains^19^.

Recent advancements in three-dimensional (3D) imaging led researchers to explore the potential of surface distance analysis applied on virtual bone models. Several studies have in fact investigated the use of the point-to-point distance between models as a quantitative tool to sort commingled skeletal remains, supporting traditional techniques^18,20–27^. About the re-association of articulating bones, the 3D approach was tested on the temporo-mandibular^20^, atlanto-occipital^21^ and sacroiliac joints^28^. The test on the re-association of temporal bones to their corresponding mandibles did not produce statistically significant results, hence the technique is not recommended for this purpose^20^. In contrast, the attempt to sort atlanto-occipital joints through 3D superimposition^21^ resulted in high sensitivity rates (100%), although specificity was considerably low (32%). Distance analysis of the sacroiliac joint^28^ produced similar sensitivity rates (99%) although specificity was higher (80%). Additional works considering 3D bone models, but with a different approach to the distance analyses (i.e., Procrustes analysis), focused on the re-association of femora and innominate bones demonstrating that the proposed semi-automatic landmark-based approach represented a powerful tool to sort the two skeletal elements with a high accuracy^29^.

All in all, virtual anthropological techniques provided promising results for some articular adjoining surfaces, although several joints are still to be explored. The re-association of crucial joints might gain a fundamental role in the anthropological analysis, above all allowing to reliably assess the biological profile of the individuals. Some articulations, more than others, are decisive, as for instance the atlanto-occipital and the atlanto-axial joints. These two articulations can, in fact, allow to re-assemble the entire skeleton in some forensic contexts. Indeed, the head or the cranium (depending on the state of preservation) might be the only body part disarticulated from the rest. In this case, disarticulation can occur at the atlanto-occipital level^21,30,31^ or the cranium may be found still attached to the atlas, but not to the axis. Therefore, it appears clear the importance to investigate through 3D virtual anthropological approach some of the most crucial adjoining bones. On the one hand, some efforts have been already made for the atlanto-occipital joint^21^. On the other hand, the same issue for the atlanto-axial joint is still to be solved. This study attempts at filling exactly this gap: here, the focus is on the articular facets of the atlanto-axial joint and the potential of their 3D configuration and congruency. This study therefore extends the research avenue recently started that investigates the application of 3D-3D superimposition and distance analysis to the resolution of commingled assemblages.

## Materials and methods

### Sample

The sample of the study includes 44 atlases and 43 axes from unclaimed documented individuals of the Collezione Antropologica LABANOF (CAL) Milano Cemetery Skeletal Collection^32^. In particular, fourty-one atlases (C1) and 41 axes (C2) from the same individual were selected from 18 males and 23 females aged respectively between 29 to 91 years (mean: 63 ± 22 years) and 24 to 88 years (mean: 72 ± 15 years). The size of the sample is limited to the individuals of the collection that had been processed and studied at the time of the sample selection and that complied the following inclusion criteria. (i) The presence of both skeletal elements of interest for the matching group; (ii) the good state of preservation of the two bones without taphonomic and traumatic alterations; (iii) the absence of severe pathological condition which might have modified both morphology and dimensions of the analysed bone structures; (iv) the bones belong to adult individuals. In addition, 5 unpaired vertebrae (3 atlases and 2 axes from 3 females and 2 males) were selected to simulate a real commingled context where it is unlikely that all commingled bones will have a match^18,27^.

### 3D analysis: ROIs selection and superimposition

**Figure 1:**
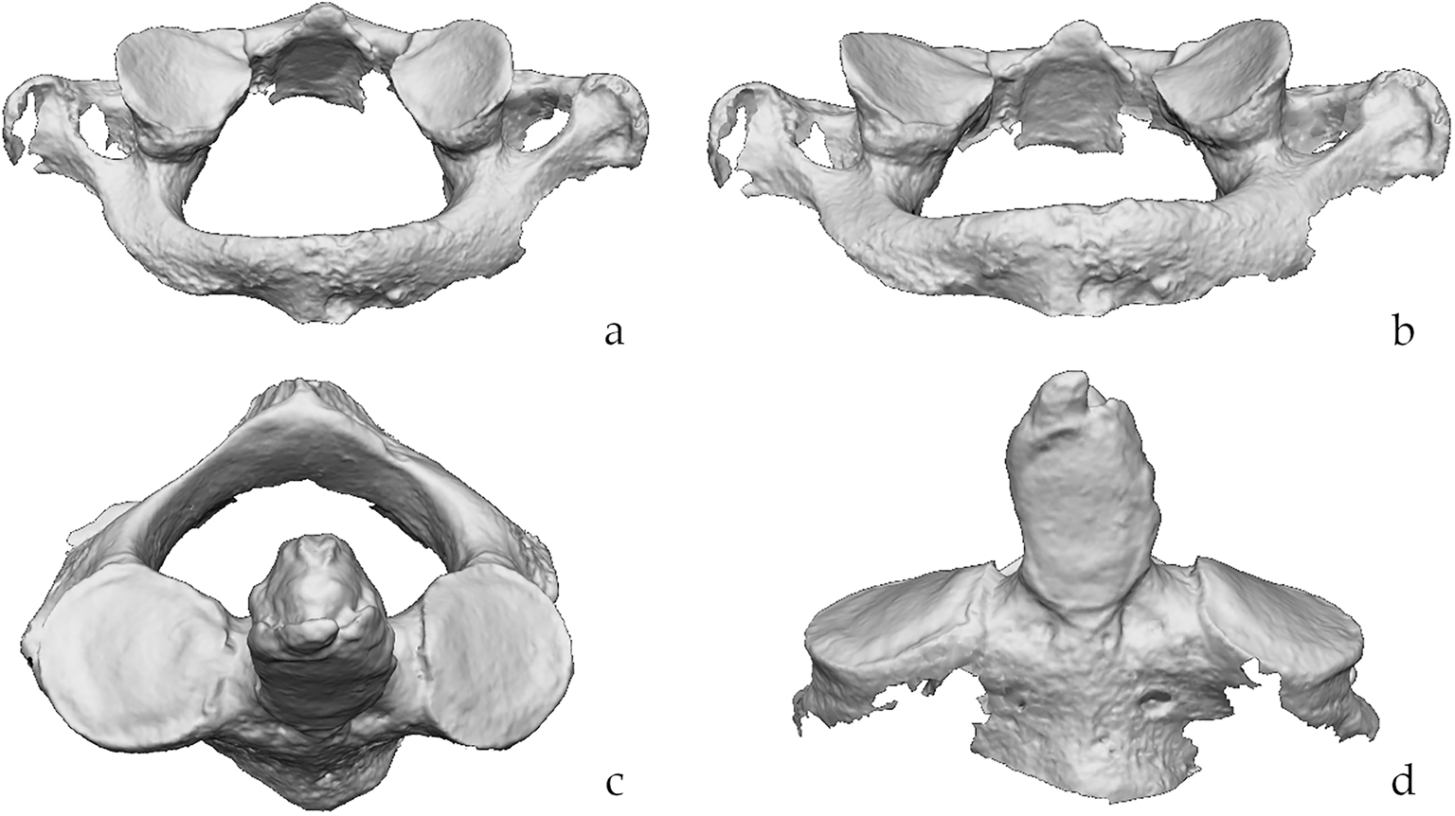
Acquisition of the models. Example of the 3D models of atlas (**a**,**b**) and axis (**c**,**d**) obtained with the laser-scanner instrument. (**a**,**b**) infero-posterior view of the atlas at different angles; (**c**) antero-superior view of the axis; (**d**) frontal view of the axis. Scale is not respected.

Three-dimensional (3D) models of the vertebrae (Fig. 1) were acquired with the Dental Wings 3Series (Dental Wings Inc., Montreal, Canada), a laser-scanning instrument with an accuracy of 15 µm. To perform the scan, the bones were positioned with the articular surfaces of interest (i.e., the inferior facets of the atlas and the superior ones of the axis) facing the light-source.

**Figure 2:**
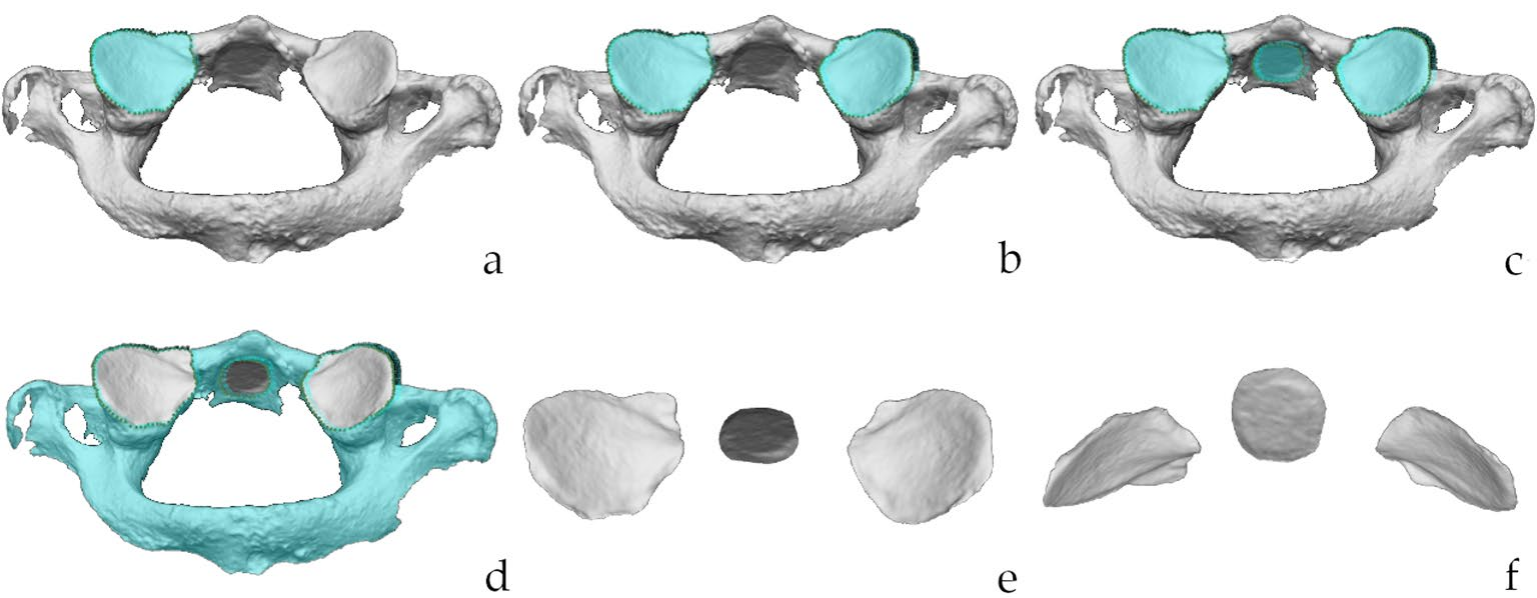
Isolation of the ROIs. Steps of the protocol for the isolation of the ROIs on the atlas (postero-inferior view): selection of the right (**a**) and left (**b**) articular surface; (**c**) selection of the articular facet for the tooth of the axis; (**d**) inversion of the selection; (**e**) deletion of the vertebra beside the articular facets; (**f**) frontal view orientation of the articular facet. Scale is not respected.

**Figure 3:**
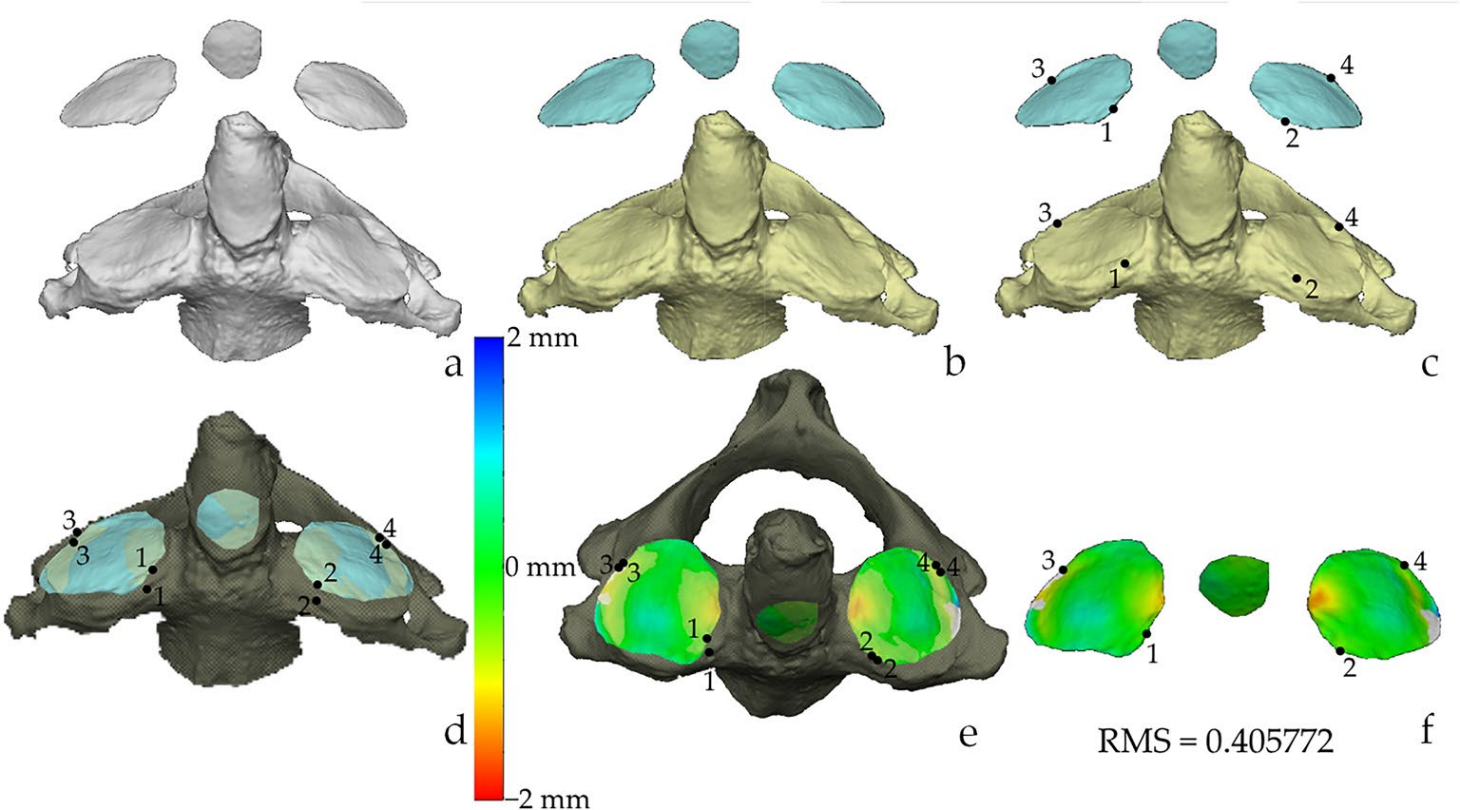
Distance analysis 1. Superimposition and calculation of the RMS value of a match. (**a**) appearance of the 3D models when opened in VAM; (**b**) coloration of the meshes for graphical purposes: axis (yellow) and ROIs of the atlas (light blue); (**c**) positioning of the landmarks on both 3D models; (**d**) landmark-based registration; (**e**) whole-surface registration through ICP algorithm and calculation of the RMS; (**f**) colour map of the superimposition. In (**d**) and (**e**) the axis is darker because “disactivated” to allow a better visualisation of the atlas. Scale is not respected.

**Figure 4:**
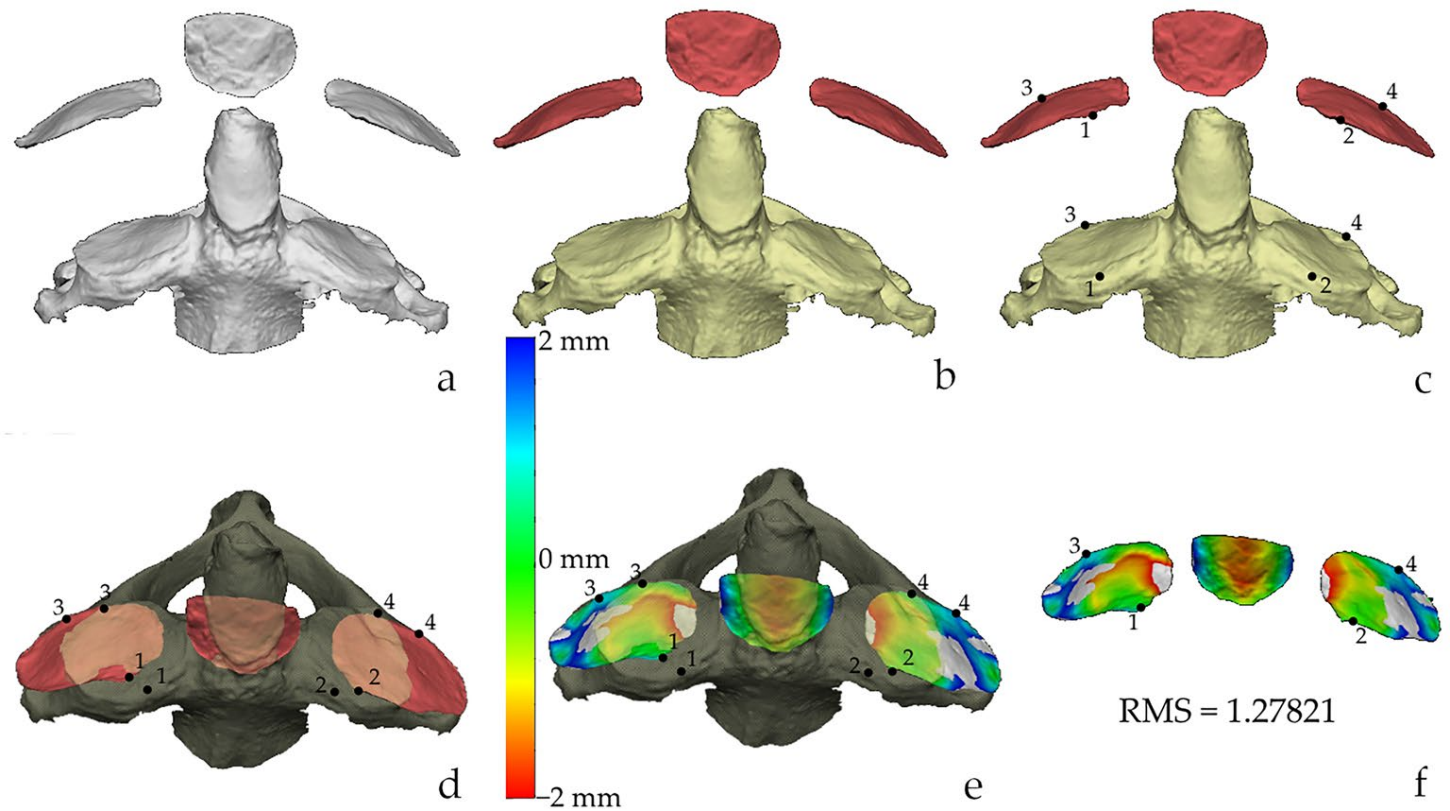
Distance analysis 2. Superimposition and calculation of the RMS value of a mismatch. (**a**) appearance of the 3D models when opened in VAM; (**b**) coloration of the meshes for graphical purposes: axis (yellow) and ROIs of the atlas (red); (**c**) positioning of the landmarks on both 3D models; (**d**) landmark-based registration; (**e**) whole-surface registration through ICP algorithm and calculation of the RMS; (**f**) colour map of the superimposition. In (**d**) and (**e**) the axis is darker because “disactivated” to allow a better visualisation of the atlas. Scale is not respected.

The procedure for the 3D analysis included three main steps which were carried out on the Vectra Analysis Module software (VAM, version 2.8.3; Canfield Scientific, Parsippany, NJ, USA): (i) the selection of the regions of interest (ROIs, i.e. articular facets of the atlas); (ii) the superimposition of the articular facets of the atlas with those of the axis; (iii) the calculation of the Root Mean Square (RMS) point-to-point distance, which is the value measuring the magnitude of the differences between two surfaces^26,33,34^. In particular, the protocol (Table 1) is a slightly modified version of the procedure for the sorting of the atlanto-occipital joints^21^. Indeed, the landmarks for the ROIs selection were placed only on the contours of the articular facets of atlases (Fig. 2), including the two inferior articular facets and the facet for the dens. Neither selection nor isolation was performed for the articular facets of axes. Conversely, the previous protocol included the ROIs selection and isolation of the articular surfaces from both bones. As a result, the selection included only the surface enclosed by the delimiting landmarks. The selection was then inverted so that it included the surrounding bone which was then deleted (Fig. 2). This approach of ROIs selection allowed the articular surfaces to keep their 3D positioning, and it was performed for all 44 atlases. The second methodological advancement compared to the original protocol concerned the superimposition. This was performed between the isolated articular surfaces of the atlas on the corresponding articular surfaces of the axis that, as highlighted for the previous step, were not isolated (Figs. 3, 4). To achieve the best possible superimposition, a two-step registration (superimposition) of the isolated articular surfaces of the atlas on the articular surfaces of the whole axis was performed. Firstly, a landmark-based registration was used to correctly orient the corresponding articular surfaces of the two skeletal elements (Figs. 3, 4), by placing two landmarks on the most antero-medial point and postero-lateral points of each articular surface (Figs. 3, 4). No landmarks were positioned on the articular surface of the atlas for the dens of the axis, and correspondingly on the odontoid process of the axis, because those of the articular surfaces were enough to reach a correct alignment between all the articular surfaces. The second registration step was the automatic one performed by the software through an ICP (Iterative Closest Point) algorithm that allow to obtain the closest point-to-point distance among the superimposing surfaces (Figs. 3, 4). Finally, the RMS (Root Mean Square) point-to-point distance (measured in millimetres) was automatically calculated for all superimpositions (matches and mismatches) by the VAM software, according to previous studies^34–37^. The software generates a colour-coded map depicting the best-fitting regions (green) and those with greater distances (blue or red) (Figs. 3, 4).

**Table 1:** Step-by-step description of the protocol and average time of completion of the steps.

Step	Passage	Procedure	Average time required
0	1	Scan one-by-one all the skeletal elements with the laser scanning instruments (Dental Wings 3Series)	To scan a single bone: 6 min
	2	Save each 3D model in .stl file format	
1	3	Open one atlas in the Vectra Analysis Module (VAM) software	To isolate ROI from one atlas: 5 min
	4^a^	Isolate the ROIs from the atlas: the two articular surfaces and the one for the odontoid process of the axis	
	5^a^	Save the isolated ROIs of the atlas	
2	6	Open the isolated ROIs of one atlas	To perform the superimposition procedure and obtain the RMS values (one atlas superimposed to all 43 axis): 47 min
	7	Import the whole axis model within the working window of the VAM software	
	8	Place in the atlas Landmarks 1 and 2 on the most antero-medial point of the right and left articular surface respectively and Landmarks 3 and 4 on the most latero-posterior points of the right and left articular surfaces respectively	
	9	Place the corresponding landmarks on the articular surfaces of the axis. Landmarks 1 and 2 on the most antero-medial point of right and left surface, and landmarks 3 and 4 on the most postero-lateral points of the right and left surfaces respectively	
	10	Perform the landmark-based registration of the two surfaces moving the atlas onto the axis. The latter is kept as the fixed element	
	11	Perform the surface-based registration automatically through the software ICP algorithm	
3	12	Calculate the RMS value and obtain the colour-coded map of the superimposition	
	13^b^	Exclude the possible superimposition because of the size-based 3D virtual assessment	
4	14	Annotate the RMS value	
	15	Close the axis, and import a new axis in the working window of the atlas (repeat steps 7–15) until the atlas has been superimposed to all axes	

^a^The ROIs selection must be performed for all atlases.

^b^The step is optional.

Following the new protocol, a “free-for-all” test was performed in which every atlas (n = 44) was superimposed on every axis (n = 43) whether they belonged to the same (“matches”) or a different (“mismatches”) individual. Indeed, the mismatches group includes superimpositions of elements from individuals of corresponding or non-corresponding sex. In light of the possible differences in gross size between bones of male and of female individuals (which may influence the results of the superimposition and of the distance analysis), the RMS values of the whole mismatching group were further investigated to determine if they resulted from a superimposition between corresponding sex (CS) or of non-corresponding sex (NCS) individuals.

The intra- and inter-operator reliability of the protocol were verified on 20 superimpositions (10 matches and 10 mismatches) performed twice by the operator 1 (R.S.) with a minimum one-month interval between sessions, and once by a different operator (A.C.).

A visual virtual 3D analysis was performed on a subset of 1010 randomly selected mismatching superimpositions. The rationale stands in exploring the possible causes for low RMS distances between mismatching vertebrae. This possibility may derive from faulty superimpositions where the Iterative Closest Point ICP algorithm could not register the surfaces correctly. To conduct the 3D virtual assessment, the degree of fitting of the articular surfaces of the atlas compared to those of the axis (and vice versa) was evaluated. In a similar fashion to the visual/manual assessment, it was noted whether there was a “size” discrepancy because one bone significantly exceeded or was enclosed by the other. These superimpositions were considered as “faulty”. In the present study, the “faulty” superimpositions corresponded to scores 4 and 5 as proposed by Litavec^28^, because of a mostly grey colorimetric map. Being this a qualitative assessment, this analysis was tested in 50 superimpositions by two operators (A.C.) and (R.S.) to verify the inter- and intra-observer repeatability.

### Blind validation test

A test was performed blindly (R.S) on a new set of 10 atlases and 10 axes from 10 individuals that were not included in the study sample. A free-for-all test was run and the established threshold value from the original sample was applied to the test the efficacy of the method (sensitivity and specificity) in a simulated small-scale commingling. In addition, the percentage of true matches falling within the first three proposed matches according to the lowest RMS value was noted.

### Statistical analysis

The statistical analyses were all performed on SPSS (IBM Corp., Armonk, NY, version 28.0), and the graphs were created in OriginPro (OriginLab Corporation, Northampton, MA, version 2021b).

The repeatability and reproducibility of the advanced proposed protocol were assessed using the Intraclass Correlation Coefficient (ICC). The ICC for the intra- and inter-operator reliability was calculated as a single measurement, absolute agreement, two-way mixed-effects model, and interpreted according to Koo and Li^38^. The intra- and inter-observer repeatability in the “faulty”/“non-faulty” categorization has been assessed using the Cohen’s kappa^39^ and interpreted according to Landis and Koch^40^.

Descriptive statistics of RMS values of matches and mismatches subdivided according to sex were obtained. In addition, descriptive data for the superimpositions of mismatches of noncorresponding sex was provided. The normal distribution and the homoscedasticity (homogeneity of variances) of the ages between males and females, and of the RMS values of matches and mismatches were tested through the Shapiro–Wilk’s test and the Levene’s test, respectively. Statistically significant differences between the age of males and females were assessed using a Student’s t-test for heteroscedastic data (Welch’s test). Statistically significant differences of RMS values between males and females and matches and mismatches groups (total, corresponding and non-corresponding sex) were assessed through Student’s t-test for independent groups in case the assumption of normal distribution was respected. Otherwise, Mann–Whitney U test was applied. In all cases, a 0.05 alpha level of statistical significance was chosen (α = 0.05).

In addition, a Receiver Operating Characteristic (ROC) curve analysis was performed to graphically illustrate the results in regard to sensitivity (True Positive Rate on the y-axis) as function of (1 − specificity) (False Positive Rate on the x-axis)^23^ of the RMS values generated by the total 1892 superimpositions. The calculation of the Area Under the Curve (AUC) allows for accurately interpreting the statistical significance of the ROC curve^41^ as it measures the success rate of a parameter in differentiating between two groups. The closer the AUC value to 1, the more discriminative is the test, whereas values equal to or below 0.5 indicate that the method is ineffective^42^. An additional summary index obtainable from the ROC curve is the Youden index^43^ which allows to determine the optimal cut-off value as: sensitivity + specificity − 1. The Youden index represents the maximum difference between the True Positive Rate to the False Positive Rate and it gives the same weight to sensitivity and specificity^44^.

### Ethical statement

The collection was assembled with individual skeletons from cemeteries of the city of Milano, in strict agreement with Italian regulations (Police Mortuary Rules-DPR 09.10.1990 No. 285, art. 43 and Regio Decreto-08.31.1933 No. 1592, art. 32) which grant universities to collect unclaimed skeletal remains for educational and research purposes. All individuals were anonymized before entering the collection. The use of the samples from the collection for this study is authorized by the abovementioned regulations^32,45^. Informed consent was not required. All methods were carried out in accordance with the Italian law, institutional guidelines and regulations.

### Institutional review board statement

This manuscript does not involve human living participants nor animals. The study follows the Police Mortuary Rules (DPR 09.10.1990 No. 285, art. 43) and the Regio Decreto (08.31.1933 No. 1592, art. 32).

## Results

The protocol for the selection of the ROIs and the superimposition of the articular surfaces of the atlanto-axial joint proved to be highly repeatable and reproducible. Intra-operator reliability ICC and the 95% CI are always above 0.90, similarly to the inter-operator even though the lower limit of the 95% CI of matches and mismatches is slightly below 0.90 in both cases (Table 2**)**. No differences according to age of males and females were observed (p = 0.098) through the Welch’s t-test. The RMS values of matches were normally distributed and homoscedastic (p > 0.05), while those of mismatches were non-normally distributed and heteroscedastic. Thus, parametric and non-parametric tests were used according to the distribution of the data. Student’s t-test showed no statistically significant differences between RMS values of matches of males and females (p = 0.270), and so did the Mann Whitney U test for the RMS values of mismatches between sexes (p = 0.210). Thus, males and females showed no significant differences in matches and mismatches allowing to pool together all RMS values. Regardless of sex, the Mann–Whitney U test showed a statistically significant difference (p < 0.001) between the two groups. Statistically significant differences were also observed when mismatches of corresponding sex and mismatch of non-corresponding sex were tested (p = 0.037), and when total matches were compared to corresponding sex (p < 0.001) and non-corresponding sex mismatches (p < 0.001). The type of superimposition and corresponding p-values are reported in Table 3.

**Table 2:** Intraclass correlation coefficients and 95% confidence interval for intra-operator and inter-operator reliability for ROIs selection.

	Intra-observer reliability		Inter-observer reliability	
	Matches	Mismatches	Matches	Mismatches
ICC	0.982	0.994	0.970	0.970
95% CI	0.914–0.996	0.975–0.998	0.891–0.992	0.886–0.993

*ICC* Intraclass correlation coefficient, *CI* Confidence interval.

**Table 3:** Significant differences (p-value) of RMS values of the evaluated types of superimpositions.

Type of superimposition	Significance (p-value)
Match_Male_–Match_Female_	0.270*
Mismatch_Male_–Mismatch_Female_	0.210^#^
Match_Total_–Mismatch_Total_	**< 0.001**^**#**^
Mismatch_CorrespondingSex_–Mismatch_NonCorrespondingSex_	**0.037**^**#**^
Match_Total_–Mismatch_CorrespondingSex_	**< 0.001**^**#**^
Match_Total_–Mismatch_NonCorrespondingSex_	**< 0.001**^**#**^

In bold statistically significant differences with significance for p < 0.05. *Student’s t-test; ^#^Mann–Whitney U test.

The superimposition of the articular surfaces of each atlas with all available axes allowed to obtain 1892 superimpositions (Table 4), 41 of which corresponded to true matches (atlas-axis joint from the same individual), and 1851 were mismatches (atlas-axis joint from different individuals). Of these 1851 mismatches, 343 were male-male mismatches and 577 were female-female mismatches, thus 920 mismatches were corresponding sex mismatches (atlas and axis are of two individuals of the same sex). The remaining 931 superimpositions were non-corresponding sex mismatches (i.e., the bones belong to individuals of different sexes). Examples of matching and mismatching superimpositions are depicted in Figs. 3 and 4, respectively.

**Table 4:** Descriptive statistics (mean, standard deviation, minimum and maximum) of the RMS values subdivided according to sex, and type of superimposition (matches or mismatches).

	Matches			Mismatches			
	Male	Female	Total	Male	Female	Non corresponding sex	Total
N	18	23	41	343	577	931	1851
Mean (mm)	0.58	0.61	0.60	0.78	0.77	0.81	0.79
Standard deviation (mm)	0.10	0.10	0.10	0.20	0.23	0.26	0.24
Minimum (mm)	0.41	0.40	0.40	0.45	0.43	0.37	0.37
Maximum (mm)	0.76	0.77	0.77	1.61	1.77	2.18	2.18

**Figure 5:**
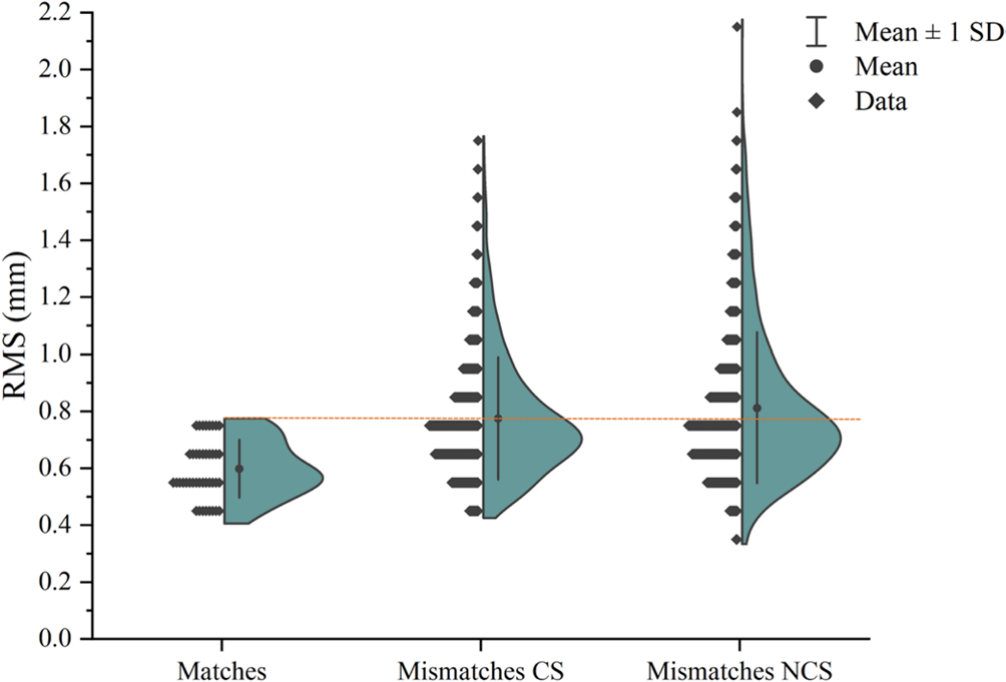
Violin plot of RMS values. Distribution of RMS values of matches, corresponding sex (CS) mismatches, and non-corresponding sex (NCS) mismatches.

RMS values of total matches ranged from 0.40 to 0.77 mm, whereas those of total mismatches ranged from 0.37 to 2.18 mm, regardless of the corresponding or non-corresponding sex. In particular, the highest RMS values corresponded to those provided from the superimposition of elements belonging to different sex. Based on the threshold of 0.77 mm, 59% of the RMS values obtained from the superimposition of the mismatches were lower than the maximum RMS values of the total matches. Figure 5 shows the distribution of RMS values of matches, corresponding sex (CS) mismatches, and non-corresponding sex (NCS) mismatches with related mean, standard deviations and minimum and maximum values. The peak of the CS and NCS mismatches’ curves is at RMS = 0.70 mm and represents 41% (376 out of 920) and 39% (360 out of 931) of the superimpositions, respectively. Above the threshold value (0.77 mm) only the excluded mismatches: 39% (358 out of 920) of CS mismatches, and 44% of NCS mismatches (406 out of 931). As for the unpaired vertebrae, they generated 217 superimpositions of which only 26 were false positives representing 12% of this subset and 2% out of the total 1087 false positives.

**Figure 6:**
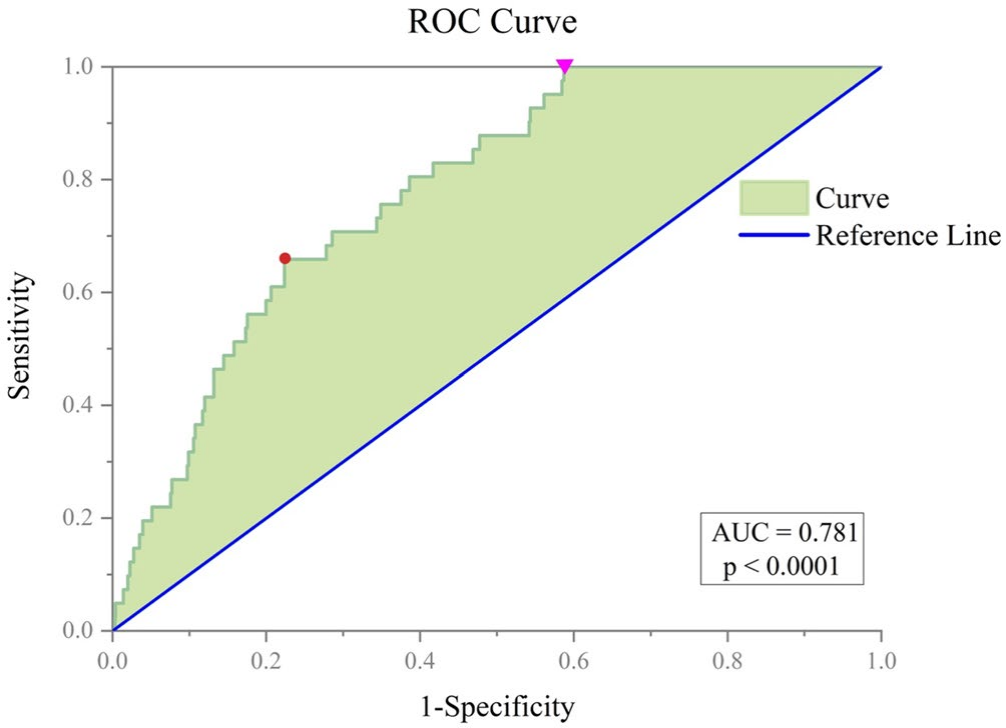
ROC curve analysis. ROC curve plot: the red point indicates the optimal cut-off according to the Youden index. The purple triangle is the cut-point corresponding to the threshold (100% sensitivity) set by the authors.

The ROC curve analysis (Fig. 6) of the 1892 superimpositions (41 matches, 1851 mismatches) produced an AUC value of 0.781 (95% IC 0.724–0.838; standard error: 0.029; p-value < 0.0001). This value of AUC value is interpreted as “fair” according to Nahm^42^. Therefore, the RMS value of 0.77 mm is a fair threshold for excluding non-corresponding vertebrae. The theoretical optimal cut-off value, determined according to the Youden index^43^, would be an RMS value of 0.62 mm that would lead to 66% of sensitivity and 78% of specificity.

For the reanalysis of the 1010 mismatching vertebrae, 554 RMS distances corresponded to false positive values, according to the 0.77 mm threshold. The intra- and inter-operator reliability for the virtual visual 3D assessment to categorize faulty and non-faulty superimpositions was both reproducible (K = 0.864, p < 0.001) and repeatable (K = 0.831, p < 0.001) with an “almost perfect agreement” according to Landis and Koch^40^. The virtual visual 3D assessment showed that 443 (80%) of the false positives resulted from faulty superimpositions given by incongruous surfaces that exceeded or were enclosed one into the other. The remaining 20% were false positive values resulting from the superimposition of surfaces whose congruency could not be excluded by eye.

### Blind validation test

**Figure 7:**
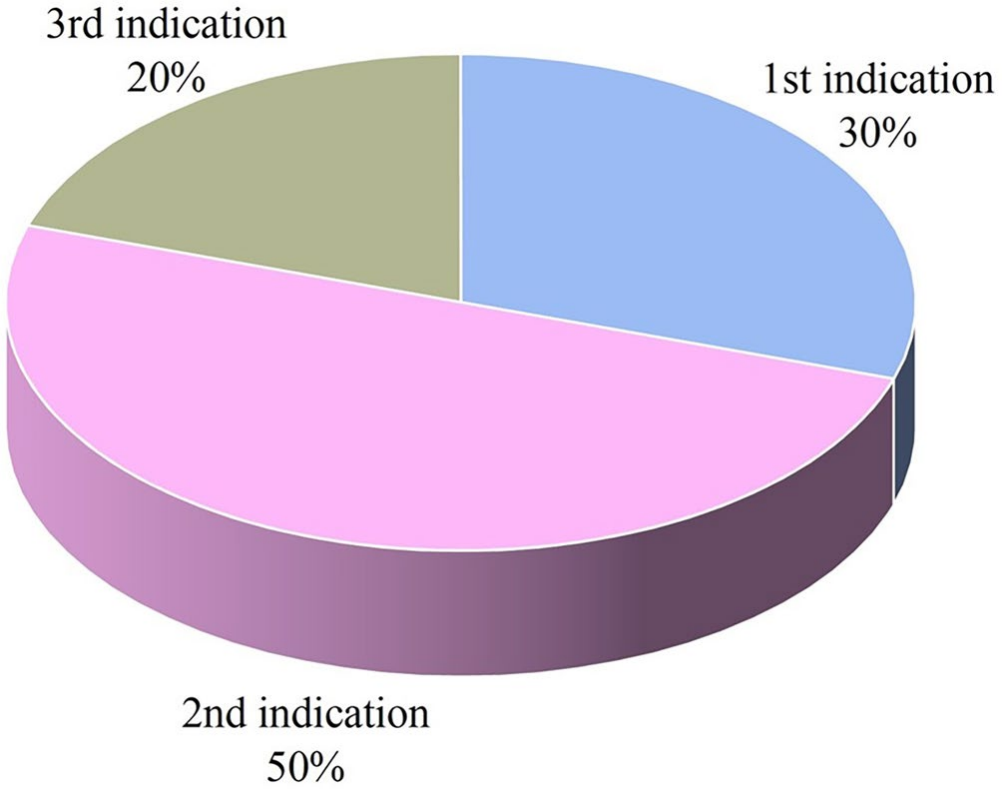
Correct sorting rate for the matches included in the blind validation test. First indication is considered to be the superimposition between the atlas with the axis that showed the lowest RMS, second indication is the second lowest RMS and third indication the third lowest RMS value. All matches’ superimpositions provided RMS distances in the first three lowest values.

The validation test included 100 superimpositions between vertebrae, of which 10 matches and 90 mismatches. The validation test confirmed the results deriving from the original sample: all matches had an RMS value below the threshold value of 0.77 mm (sensitivity: 100%). Thirty-four out of 90 mismatches were above the threshold value (specificity: 38%). The correct match was indicated either by the first, second or third lowest RMS value. Specifically, 30% of the correct matches corresponded to the first indication (lowest RMS value), 50% and 20% of the matches were indicated by the second (second lower RMS value) and third indication (third lower RMS value), respectively (Fig. 7).

## Discussion

The resolution of commingled assemblages is a challenging task that anthropologists started to systematically explore in the last decade. However, it is not an uncommon finding especially in the aftermath of mass disasters^30,46^, conflicts^5,47,48^, or in archaeological settings^49^, where impressive amounts of commingled remains are recovered. Together with pair-matching of bilateral bones, articulation of adjoining elements serves to reassemble individual skeletons. By re-fitting portions of the vertebral column, anthropologists can re-build almost the entire skeleton, or at least assemble anatomical districts that are paramount to produce a sound biological profile. Indeed, some articulations can allow to re-associate the cranium to the post-cranium which is fundamental for a proper profiling of the remains, particularly in those contexts where the skull is the only element disarticulated from the rest of the body^21^. When the disarticulation occurs at the atlantoaxial joint instead, it is evident that correctly matching atlas and axis may facilitate to assemble the entire skeleton and create a complete biological profile of the individual.

Recently, 3D–3D superimposition and distance analysis have been tested in the sorting and re-association of commingled remains, especially for the pair-matching of bilateral elements^18,22,24,26,27^. Moreover, the technique was applied to the re-association of adjoining bones, with variable results^20,21^. Generally, this kind of approach allows to detect the maximum RMS value that a correct association hypothetically might reach, and thus its selection as discriminative threshold value should enable 100% sensitivity. The specificity depends instead on other logics and factors and might result greater or lower depending on the articulation under analysis and its overall morphological configuration. Although in the present study the chosen threshold of 0.77 mm produced unbalanced sensitivity and specificity rates, with 59% of false positives values below the threshold, it represents the ideal cutoff to detect all the true positives (matches), hence reaching 100% sensitivity rate. In a forensic context, one would rather screen the sample to determine all possible matches that would be further investigated by other means. Indeed, these rates seem a reasonable compromise in certain forensic contexts where the requirements are analogous to those pertaining diagnostic and clinical ones. As demonstrated by the ROC curve analysis, the optimal cut-off value according to the Youden Index, a summary measure of the discriminatory effectiveness of the method, would correspond to a threshold of 0.62 mm with 66% of sensitivity and 78% of specificity. In a forensic case, missing such a high number of true matches would not be acceptable. Therefore, the authors would advise including a higher number of false positives, rather than misidentifying a true match. As a result, a 100% sensitive threshold should be preferred even at the detriment of specificity.

Even though the accuracy is not optimal, the 3D method applied to this joint performed better than previous test on the atlanto-occipital articulation, where only 32% of the mismatches had an RMS distance value above the threshold^21^. Here, in addition to the inferior articular facets of the atlas and the superior ones of the axis, the facet for the dens was included: the third articular surface thus participates in the superimposition and calculation of the RMS distance value which may have contributed to a higher exclusionary power of the method. Both inter-observer and intra-observer repeatability can be classified as excellent^38^, being higher than in the re-association of crania and atlases^21^, possibly because the models were acquired with the same device (i.e., laser scanner), whereas in the previous study, two technologies were used, namely laser scanner for the atlas and stereophotogrammetry for the occipital condyles. The use of two different instruments may have caused the slight decrease in the repeatability values. Moreover, the protocol used by the present study was slightly different: here, only the ROIs of atlases were isolated and then superimposed on the articular facets of entire axes, while in the previous study all the articular surfaces, both the occipital condyles and the atlas superior articular surfaces, were selected and then superimposed between each other, which may have contributed to increase the variability between observations. Another difference with the previous study is the inclusion of unpaired elements in the present sample to simulate a commingled scenario where the anthropologists do not know ab initio whether all matching bones are present. Unpaired bones generated only the 2% of false positive values, compared to the whole sample of 1087 mismatching vertebrae. As such, the presence of unpaired elements does not represent a significant hindrance to the sorting method, since 90% of the RMS values from unpaired elements were correctly identified as true negatives.

A further investigation into the high number of false positive values (i.e., mismatches with RMS distance values below the threshold) was performed. The RMS distance values deriving from the large number of superimpositions of mismatches (1851 in total) ranged between 0.37 and 2.17 mm, and specifically the 59% of these values are false positives, being values below the threshold one (0.77 mm). The authors wondered whether the low RMS values of false positive were produced because of a higher similarity/congruency between articular surfaces even if from different individuals. More likely, the authors suspected that the superimposition technique applied to re-association of articular surfaces presented pitfalls that jeopardize the discerning ability of the method. For instance, could a low RMS value of false positives be the result of an improper overlap of the surfaces? This may be due to the approximation of the ICP algorithm applied to meshes of articular structures that do not actually touch each other or to very limited overlapping areas of the two surfaces that do not superimpose properly because extremely different in the three dimensions, a critical point to ascertain. To this purpose, a subset of 1010 mismatching models was reevaluated by 3D visually assessing the congruency of the surfaces. It was noted that 55% of this subsample had false positive RMS values. When the superimposed articular facets of one vertebra exceeded or were undersized compared to the other vertebra, the superimposition was considered faulty. This applied also when only one surface was correctly superimposed and the contralateral was automatically displaced. Out of 554 false positive values, 80% derived from faulty superimpositions where the software did not match properly the articular surfaces between each other according to their three-dimensional anatomical orientation and congruence. This 3D virtual visual reassessment by eye further allowed to exclude non-corresponding vertebrae within the pool of false positive RMS values. Although this step could be performed manually without the 3D virtual acquisition, the isolation of the articular facets through the VAM software aided in the evaluation of the congruency as it allowed to visualize the correspondence of the articular margins. Moreover, this additional step strengthens the common belief that the sorting of commingled remains should include several approaches that integrate each other. As a result, the intrinsic limitations of the 3D–3D superimposition and of the ICP algorithm in the simulation of articular surfaces may be explored further to understand the real applicability of this technique to the re-association of joints in commingled settings.

Most of these defective superimpositions derive from elements of different sex groups (e.g., female atlas on male axis or vice versa) which may present incongruences in dimensions and distances between articular facets. This entails that the initial screening, before approaching to the 3D analysis, should also consider the gross morphology in order to exclude visually those comparisons between highly incongruent elements that would improperly overlap and provide low RMS values. Indeed, the use of ICP algorithm for the superimposition of articular surfaces may lead to the intersection between the two surfaces rather than their adequate overlap. Therefore, the intersection between two superimposed surfaces or the presence of very limited areas possibly superimposing between two incongruent surfaces are both factors contributing to low RMS values, thus hindering the real ability to discriminate between matches and mismatches. Hence, especially in a forensic scenario, this represents a considerable issue and an intrinsic limitation of the approach that needs to be acknowledged and then improved in the future advancements of 3D virtual techniques. Nevertheless, the contribution of this approach to the progress of commingled remains analysis is unquestionable. Hence, as a possible solution for avoiding misinterpretations of results, we caution on the ICP algorithm superimposition procedure and to check its quality or rather exclude any improper overlapping. Even though no significant differences of RMS values between sexes were recorded, data were presented separately. This could be beneficial in cases where highly dimorphic bones are articulated to the vertebrae that have to be sorted (e.g., an atlas attached to the respective cranium, or an axis associated to the rest of the column and the pelvis). Moreover, when only one sex group is represented in the commingled assemblage^50^, a sex-specific threshold may allow a more accurate sorting of the remains based on this approach. Although some attempts to estimate skeletal sex from cervical vertebrae were made^51–53^, this issue remains challenging and requires further research. As a result, the gross size and fit of the surfaces remain the first criteria for the initial sorting of the bones to be further evaluated. Despite the possible variations related to the anatomical configuration of the vertebrae, the method presented here serves a screening test that excludes a significant amount of RMS values above the threshold, with a high degree of confidence. This ability was further investigated in the blind validation test which yielded similar specificity rates to the study sample, supporting the exclusionary power of the method. Most times, the correct match was included in the first three lowest RMS values. This allowed to considerably reduce the possible combinations of matching atlases and axes. Indeed, the sorting based on anthropological criteria can be considered as a preliminary procedure that facilitates subsequent analyses for the re-association of the remains, such as DNA testing^2,18,23,26,31,48^.

Recently, 3D–3D superimposition has been successfully applied to the pair-matching of commingled remains^18,23,26,27^. Compared to these studies, the 3D re-association of joints yielded considerably lower results as for specificity. If unaffected by pathologies or traumas of various nature, bilateral elements present roughly specular features, although they may differ due to asymmetry^23^, whereas joint surfaces may present different shapes and features. Reports on the temporomandibular^20^ and atlanto-axial^21^ joints produced generally less efficient results, in contrast the sacroiliac joint for which high sensitivity and specificity rates were recorded by combining quantitative and qualitative parameters^28^. The planar structures of the articular surfaces of the vertebrae are virtually featureless, possibly hampering the successful application of the 3D method for the sorting of commingled vertebrae. It is therefore worth bringing into question the strength and effectiveness of this technique for such a purpose. Geometric morphometric (GMM) analysis may represent a valuable addition to the novel set of methods for the re-association of adjoining bones, as recently reported for the hip joint^29^. Following the GMM process of Procrustes superimposition, the authors enhanced the accuracy of their exclusion rate which ranged from 94 to 100% in large and small-scale commingled context, respectively. Given that the hip joint is an enarthrosis with a complex three-dimensional anatomical structure, analyzing it using 3D virtual approaches yields more satisfactory results compared to diarthroses like vertebral articulations. The differences in anatomical structures between these joints may explain the variation in results between Anastopoulou’s study^29^ and ours. However, the discrepancy in exclusion rates between their study and ours may also stem from differences in methodological approaches. By employing explanatory multivariate analyses, such as those offered by GMM, potential limitations associated with single scalar summary statistics can be overcome, opening up new and promising avenues for 3D analyses of planar articular surfaces like those found in vertebrae.

The few studies on 3D virtual approaches for the re-association of joints represent only the starting point. Further research for this purpose is necessary to confirm whether we are headed in the right direction or if we must recognize the inadequacy of the 3D virtual methodologies for re-associating the adjoining skeletal elements based on the non-optimal specificity obtained in most of the studies. The main advantage of 3D–3D superimposition relies on the numerical value provided by the comparison, which quantifies the congruency between the articular surfaces under examination. In addition, digitalization provides several benefits, including availability and accessibility to the models, preservation of the specimens and their features, creation of osteological datasets, development of methods, request for experts’ opinion^54–56^. Nevertheless, this study presents limitations. The number of bones included in our sample provided only a limited number of true matches, despite the large number of mismatches. Thus, the threshold we are suggesting is based solely on 41 matches superimpositions, stressing the preliminary nature of our study and the need for caution when applying the exclusionary 3D approach to real contexts. Again, we wish to point out that the threshold individualized from the original sample, although confirming 100% of sensitivity in the new subsamples used for the blind test, needs further validation tests on larger sets deriving also from diverse populations. In particular, although similar to previous studies^21^, the sample was limited to 87 bones from 46 individuals of the CAL collection. The sample was created sourcing the set of skeletons that had been processed and studied at the time of the sample selection and that respected the inclusion criteria (see Materials and Methods). This prevented from creating a larger set of vertebrae, which could be expanded in further tests that would clarify the performance of the method in assemblages with more commingled bones and of diverse geochronological populations, opening gap for further research opportunities. Furthermore, according to the authors who developed the original method^18^, this virtual approach is independent from sex, chronology and population group of the specimens, representing a significant improvement compared to osteometric models. However, for accomplishing such 3D virtual methodology in further validating research or in real forensic cases, anthropologists should be equipped with devices and software and possess the necessary expertise demanded for the application of such protocols, a limitation not of a trivial worth. Overall, the reproducibility of such 3D approaches is still an objective to be considered and fully verified: could a different scanning device produce different results? This is a further important methodological aspect that still needs to be investigated and may represent a potential new research question to be tested before including 3D approaches in forensic cases.

Finally, the authors would like to suggest caution before using this approach as further analyses are required to test the applicability to real forensic cases and to clarify the contribution that such methodologies may provide in addition to the traditional osteometric and visual approaches in commingled contexts.

## Conclusions

The present study investigated the re-association of atlas and axis through 3D approach proving that a high level of 3D congruency exists between their articulating surfaces and can potentially be used for re-association purposes in commingled contexts. However, based on the results, we cannot consider the 3D superimposition of the atlanto-axial articular facets as a re-association method per se, but rather as a screening method with an ideal sensitivity when applied to forensic contexts. 3D virtual methodologies applied to the atlantoaxial joint proved to perform better in comparison with other articulations previously analyzed, such as the atlanto-occipital and the temporo-mandibular joints. Further 3D virtual studies may include other skeletal portions to provide answers to the complex issue of commingled remains and to properly guide the research on the re-association of articular bones through these promising analyses.

## Data Availability

Data are available upon request.
